# Early emotional and behavioural problems predict use of habilitation services among children: Findings from a longitudinal follow-up study

**DOI:** 10.1371/journal.pone.0303685

**Published:** 2024-05-16

**Authors:** Pavithra Ashok, Anna Fäldt, Anton Dahlberg, Natalie Durbeej

**Affiliations:** Child Health and Parenting (CHAP), Department of Public Health and Caring Sciences, Uppsala University, Uppsala, Sweden; Children’s Hospital of Eastern Ontario (CHEO), University of Ontario, CANADA

## Abstract

**Purpose:**

To explore the association between early emotional and behavioural problems and use of habilitation services among children in Sweden.

**Methods:**

In this longitudinal cohort study, we used data on children, 3–5 years of age, whose mothers (n = 7343) and fathers (n = 6322) had responded to the Strengths and Difficulties Questionnaire (SDQ) for assessment of emotional and behavioural problems, and who were followed for approximately 6.5 years with regard to use of habilitation services. The relations between emotional and behavioural problems and use of habilitation services were explored through cox regression models.

**Results:**

In unadjusted models, children with identified emotional and behavioural problems were more likely to utilise habilitation services compared to those with no identified problems. These associations were shown for both mothers’ (HR: 5.02) and fathers’ (HR: 4.25) SDQ ratings. In adjusted cox-regression models, the associations remained significant for both mothers’ (AHR: 4.24) and fathers’ (AHR: 4.03) ratings.

**Conclusions:**

Early emotional and behavioural problems predict later habilitation service use among children in Sweden. Assessment of these problems in all children at child health services could facilitate early identification and timely interventions. Habilitation centres in Sweden could integrate mental health care into the standard treatment for children using these services.

## Introduction

Mental health problems among children constitute a significant public health concern. These problems affect around 10–20% of all children [1], and present a wide array of symptoms and varying degrees of severity. Emotional and behavioural problems are some of the most common mental health problems in young children [2], affecting approximately one in eight preschool children [3]. Early identification of individuals in need of support for said problems is important for promoting healthy child development.

Emotional problems in young children refer to difficulties in experiencing, expressing, and regulating emotions in a way that is appropriate to their age and developmental stage [4, 5]. These problems can manifest as excessive fear, sadness, anxieties or worries, significantly impacting the child’s daily functioning and interpersonal relationships. Behavioural problems encompass challenges in regulating behaviours according to social norms, including externalising issues like aggression, defiance, and hyperactivity, as well as difficulties with attention and impulse control. These problems are directed outward and can negatively affect others in the child’s environment. Emotional and behavioural problems are highly prevalent in children with autism spectrum disorder, attention-deficit hyperactivity disorder, intellectual disabilities, and developmental language disorder, significantly affecting their daily lives and development [6]. The phenotypic overlap between autism and emotional and behavioural problems suggests that it is important to assess both types of problems for more accurate diagnoses and interventions [7]. Further, children with developmental

disabilities display more behavioural problems than their typically developing peers [8, 9].

Studies indicate that emotional and behavioural problems are stable over time, even when assessed at an early age [10]. Further, higher levels of emotional and behavioural problems in childhood are associated with future psychopathology, including anxiety, depression and somatic health issues, as well as relational problems, substance abuse, poor academic performance, unemployment and criminal behaviour [11–15]. Identifying early signs of emotional and behavioural problems and promptly applying interventions has the potential to mitigate the challenges faced by many families. Introducing standardised and resource-efficient approaches such as questionnaires that possess suitable psychometric qualities, could aid in detecting and preventing escalation of these problems. Moreover, early interventions may positively affect children’s mental well-being in formative years, enhancing engagement in school, families, and society, and lead to lower health-related costs [16].

In Sweden, the child health services (CHS) constitute a vital part of the primary care for children as almost all children visit these services [17]. The CHS aims to promote health, monitor children’s development, and provide parenting support, making them an excellent arena for early identification of emotional and behavioural problems. However, no screening method for detection of such problems is currently used at the national CHS level. The CHS refer children for further interventions and assessment to other health care services such as speech and language pathologists (SLP), paediatricians or psychologists. When there are indications of disabilities, such as intellectual or motor impairments, children are referred to habilitation services for additional assessments and interventions. In many regions of Sweden, a specified neurodevelopmental diagnosis of for instance autism spectrum disorder is required prior to referral to the habilitation services. In the Swedish Region Uppsala, a diagnosis of autism spectrum disorders is set at these services. The habilitation services offer interventions to families of children with intellectual disabilities, autism spectrum disorders, acquired brain injury, and motor disabilities. Children with motor disabilities are commonly identified early and receive timely habilitation interventions.

According to previous research, boys, younger children, and children of parents with lower educational levels are more likely to use habilitation services than girls, older children and children of parents with higher educational levels, respectively [18–20]. In addition, children of ethnic minority parents might underutilize these services due to parental cultural beliefs, stigma or language barriers [21, 22]. At the same time, children of ethnic minority parents have demonstrated elevated risks for both emotional and behavioural problems as well as autism spectrum disorders, thus displaying great need for these services [23, 24]. Moreover, emotional and behavioural problems have been associated with lower parental educational levels [25] and gender, where boys show higher levels of disruptive behaviours and girls more symptoms of worry and anxiety [25, 26]. Also, emotional and behavioural problems have been associated with the child’s age as such problems might display different development pathways across time [26].

As far as we are aware, no Swedish studies have investigated the relation between early emotional and behavioural problems and subsequent use of habilitation services among children. Exploring this association could add knowledge on the usefulness of screening for emotional and behavioural problems, which in turn could facilitate delivery of early interventions and support to those in need. This study aimed to investigate the association between early emotional and behavioural problems and use of habilitation services among children in Sweden. Based on previous international research, we hypothesized that early emotional and behavioural problems would be positively associated with later use of habilitation services in this population [27].

## Methods

### Study design

The current study adopted a longitudinal cohort study design using data from the Children and Parents in Focus study (in short the Focus study). The Focus study aimed to investigate the health and well-being of preschool children and their parents, and to evaluate the effects of a parenting programme. It comprised a cohort of children aged 3–5 years for which data were collected in Uppsala Region, Sweden, between 2013 and 2017 [28]. In the current study, the cohort was followed with regard to registry data from 2013 to 2021, collected from Uppsala Region.

### Participants and procedure

Parents of all children aged 3 to 5 years were recruited from child health centres (CHC) in Uppsala Region during their child’s visit to the health centre for the annual health check-up visit. The nurses from the CHC affixed questionnaires to the appointment reminder letter that were routinely mailed out to every child’s parents 3 weeks prior to the annual check-up. Both parents were instructed to complete one questionnaire each and bring the questionnaires to their child’s visit to the CHC. The questionnaires included the Strengths and Difficulties Questionnaire (SDQ) for assessment of the child’s emotional and behavioural problems [29], items on child demographics, and items on parents’ socio-demographic background. The questionnaires were available in multiple languages including Swedish, English, Arabic, and Somali.

**Fig 1:**
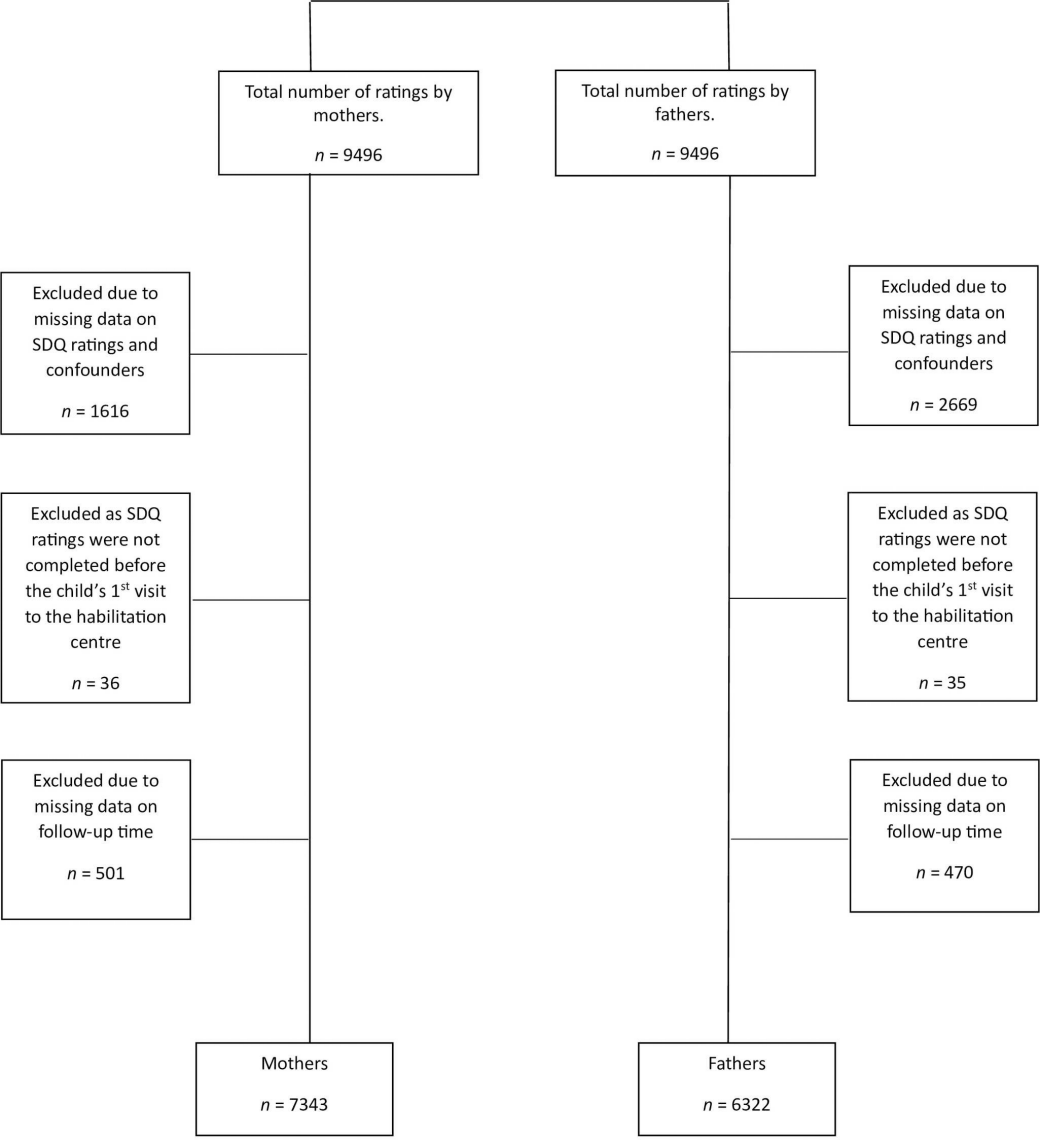
Participant flow-diagram.

Data were collected over a 4-year period (2013–2017) and at 3, 4, and 5 years of age. For children that were represented more than once, the earliest data point was selected for this study, due to our focus of exploring emotional and behavioural problems in the children when they were at their youngest age. During the study period, a total number of 9496 unique children from Uppsala Region were enrolled in the Focus study, which comprised ratings from mothers and fathers separately. The total participation rate in the Focus study was 48% in study year 1 (2013–2014), 45% in study year 2 (2014–2015), 51% in study year 3 (2015–2016), and 50% in study year 4 (2016–2017) [30]. Among the children enrolled in the Focus-study (n = 9496), we excluded children with missing data on the SDQ and potential confounders (see below) selected for this study. In addition, children whose SDQ ratings were not completed before the child’s first visit to the habilitation centre and whose SDQ ratings did not have a valid assessment date, thus yielding missing data on their follow-up time, were excluded. A total of 7343 and 6322 unique children who were rated by mothers and fathers, respectively, were consequently selected as the study samples of the current study (Fig 1).

### Data sources

#### Emotional and behavioural problems

Children’s emotional and behavioural problems were assessed using the parent version of the Strengths and Difficulties Questionnaire (SDQ) [29]. This instrument covers 25 items that are rated according to a 3-point scale, ranging from 0 to 2 (0 = Not True, 1 = Somewhat True, and 2 = Certainly True). The items can be divided into the following subscales: emotional symptoms, conduct problems, peer relationship problems, hyperactivity/inattention, and prosocial behaviour. Each subscale score ranges from 0 to 10. Additionally, the four symptom subscales (all except prosocial behaviour) can be summed into total difficulties score ranging from 0 to 40. The SDQ is one of the most widely used instruments for assessing emotional and behavioural problems in children, and has been subject to research for several decades and in many parts of the world [31–33]. Overall, assessments of the psychometric properties of the SDQ indicates good construct, concurrent and predictive validity [34–37]. The Swedish version of the SDQ has shown good construct validity, concurrent validity, and internal consistency overall [38, 39]. Thus, for this study, the SDQ was considered a useful instrument for measuring emotional and behavioural problems. We used Swedish SDQ norms available for preschool children, as rated by parents, to assess these problems [40].

#### Use of habilitation services

Data on use of habilitation services were collected through the electronic patient record system from Uppsala Region. These records include all visits to habilitation services in Uppsala Region and have demonstrated high coverage in previous research [41]. The data comprised information on the children’s date of visit to their respective habilitation service centre and concerned the period from 2013 to 2021.

#### Confounders

We included confounders pertaining to parental socio-demographic factors and child demographic factors. These data were collected through separate items from the Focus-study and included: parental country of birth (Sweden or other), parental level of education (higher/lower level), child gender (boy/girl) and child age (three/four/five years of age). This decision was based on previous studies showing associations between such factors and both emotional and behavioural problems and use of habilitation services in young children [18–26].

### Statistical analyses

Means, standard deviations, ranges, frequencies and proportions were used for descriptive purposes. The proportion of early emotional and behavioural problems among children using habilitation services compared to non-users was explored by chi-square tests. The relations between emotional and behavioural problems and use of habilitation services were explored through unadjusted and adjusted cox regression models of time to first visit to a habilitation centre, with months as the underlying time scale. The start date was the date of when the SDQ was completed by both mothers and fathers respectively and the end date was the date of the first visit to the habilitation centre or 31^st^ of December 2021 if no habilitation visits occurred. Proportional hazards assumptions were evaluated graphically by Kaplan-Meier curves, showing that the hazards were proportional for the SDQ total difficulties and the prosocial behaviour scales, but not for the remaining SDQ scales.

The cox regression models were performed in two blocks. First, emotional, and behavioural problems as measured by the SDQ total difficulties score were entered as the main exposure. Second, the SDQ lack of prosocial behaviour score along with the confounders were added to adjust the results for these variables and to separately explore their relations to the outcome. Sex- and age-specific Swedish cut-offs were applied to establish whether or not the children had identified emotional and behavioural problems as well as lack of prosocial behaviour [40]. For the SDQ total difficulties score, we used the following cut-offs for girls: ≥ 12 points (3-year-olds), ≥ 11 points (4-year-olds), and ≥10 points (5-year-olds), and boys: ≥ 13 points (3- and 4-year-olds) and ≥ 12 points (5-year-olds), respectively. For the lack of prosocial behaviour scale, we used the following cut-offs for girls: <6 points (3-,4- and 5-year olds) and boys: <5 points (3- and 4-year-olds) and <6 points (5-year-olds), respectively.

The outcome variable was defined as having at least one visit to a habilitation centre during the follow-up time (yes/no). As past research based on data from the Focus study has indicated variations in parents’ SDQ assessments of their children, separate models were employed to analyse both mothers and fathers [42]. Prior to computing the regression models, data were checked for multicollinearity between all independent variables by ensuring that the variance inflation factor (VIF) was less than 10, and none of the variables exceeded a VIF of 10 [43]. The results of the regression models are presented as crude and adjusted hazard ratios (HRs and AHRs), along with respective 95% confidence intervals (CIs). To indicate statistical significance, p- values < 0.05 were used. The statistical software used was IBM SPSS version 28.

### Ethical considerations

Parents or legal guardians of all participating children gave their written informed consent on behalf of their children, prior to inclusion in the study. The study was approved by the Regional Ethical Review Board in Uppsala/The Ethics Review Authority (document numbers 2012/437 and 2022-01389-02).

## Results

### Participant characteristics

The samples were evenly distributed in terms of gender (Table 1). About half of the participants were three years old. Most mothers and fathers were born in Sweden (84.6% and 85.3%) and had higher educational levels (72.7% and 63.6%). About 10% and 13% of the children had identified emotional and behavioural problems as rated by mothers and fathers, respectively. In addition, about 5% and 13% were identified as displaying lack of prosocial behaviour. The mean follow-up time was approximately 80 months. A total of 96 children had been using habilitation services during follow-up. The mean number of visits to a habilitation centre ranged from 1 to 162 visits in total.

**Table 1:** Description of child demographics, parental socio-demographics, children’s emotional and behavioural problems and use of habilitation services.

	Mothers’ responses (n = 7343)	Fathers’ responses (n = 6322)
Variables	n (%)	n (%)
**Child Gender**		
Boy	3745 (51.0)	3210 (50.8)
Girl	3598 (49.0)	3112 (49.2)
**Child age**		
3 years	3526 (48.1)	3124 (49.4)
4 years	2067 (28.1)	1726 (27.3)
5 years	1750 (23.8)	1472 (23.3)
**Parental country of birth**		
Sweden	6210 (84.6)	5394 (85.3)
Outside Sweden	1133 (15.4)	928 (14.7)
**Parental education**		
Lower educational level^1^	2005 (27.3)	2301 (36.4)
Higher educational level^2^	5338 (72.7)	4021 (63.6)
**Emotional and behavioural problems**		
Identified problems^3^	739 (10.1)	806 (12.7)
Mean total difficulties score (SD)	6.07 (4.27)	6.62 (4.35)
**Lack of prosocial behaviour**		
Identified problems^4^	372 (5.1)	814 (12.9)
Mean prosocial behaviour score (SD)	8.22 (1.69)	8.01 (1.77)
**Habilitation services use during follow-up**		
Mean follow-up time in months (SD, min-max)^5^	79.6 (15.9, 0.3–109.5)	78.6 (15.8, 0.3–109.3)
Use of habilitation services^6^	96 (1.3)	96 (1.5)
No use of habilitation services^7^	7247 (98.7)	6226 (98.5)
Mean no of visits to a habilitation centre (SD, min-max)	34.0 (35.4, 1–162)	31.8 (32.9, 1–162)

1 Not completed primary school/completed primary school/completed secondary school

2 College/university degree

3 Proportions of participants scoring above cut-off for the SDQ total difficulties score

4 Proportions of participants scoring above cut-off for the SDQ lack of prosocial behavior scale

5 Mean time from SDQ assessment to first habilitation centre visit

^6^ At least one visit to a habilitation centre during follow-up

^7^ No visit to a habilitation centre during follow-up

### Associations between emotional and behavioural problems and use of habilitation services

The proportions of boys, according to both mothers’ and fathers’ ratings, were larger among children using habilitation services (84.4%), compared to those not using these services (50.6% and 50.3%) (Table 2). In addition, among children using habilitation services, a larger proportion had identified emotional and behavioural problems (35.4% and 37.5%) and lack of prosocial behaviour (16.7% and 19.8%), according to both mothers’ and fathers’ ratings, than those not using such services (emotional and behavioural problems: 9.7% and 12.4%, lack of prosocial behaviour: 4.9% and 12.8%).

**Table 2:** Comparisons of children using and not using habilitation services during follow-up with regard to child demographics, parental socio-demographics, and children’s emotional and behavioural problems.

Mothers’ responses	Use of habilitation services (n = 96)	No use of habilitation services (n = 7247)		
Variables	n (%)	n (%)	χ2	p
**Child Gender**			**43.35**	**<0.001**
Boy	81 (84.4)	3664 (50.6)		
Girl	15 (15.6)	3583 (49.4)		
**Child age**			0.56	0.756
3 years	47 (49.0)	3479 (48.0)		
4 years	24 (25.0)	2043 (28.2)		
5 years	25 (26.0)	1725 (23.8)		
**Parental country of birth**			0.64	0.424
Sweden	84 (87.5)	6126 (84.5)		
Outside Sweden	12 (12.5)	1121 (15.5)		
**Parental education**			2.45	0.118
Lower educational level^1^	33 (34.4)	1972 (27.2)		
Higher educational level^2^	63 (65.6)	5275 (72.8)		
**Emotional and behavioural problems**				
Identified problems^3^	34 (35.4)	705 (9.7)	**69.08**	**<0.001**
**Lack of prosocial behaviour**				
Identified problems^4^	16 (16.7)	356 (4.9)	**27.22**	**<0.001**
**Fathers’ responses**	**Use of habilitation services (n = 96)**	**No use of habilitation services (n = 6226)**		
**Variables**	**n (%)**	**n (%)**	**χ2**	**p**
**Child Gender**			**38.74**	**<0.001**
Boy	79 (84.4)	3131 (50.3)		
Girl	17 (17.6)	3095 (49.7)		
**Child age**			2.04	0.361
3 years	48 (50.0)	3076 (49.4)		
4 years	21 (21.9)	1705 (27.4)		
5 years	27 (28.1)	1445 (23.2)		
**Parental country of birth**			0.00	0.979
Sweden	82 (85.4)	5312 (85.3)		
Outside Sweden	14 (14.6)	914 (14.7)		
**Parental education**			0.71	0.400
Lower educational level^1^	31 (32.3)	2270 (36.5)		
Higher educational level^2^	65 (67.7)	3956 (63.5)		
**Emotional and behavioural problems**				
Identified problems^3^	36 (37.5)	770 (12.4)	**53.68**	**< 0.001**
**Lack of prosocial behaviour**				
Identified problems^4^	19 (19.8)	795 (12.8)	**4.16**	**0.041**

^1^ Not completed primary school/completed primary school/completed secondary school

^2^ College/university degree

^3^ Proportions of partaicipants scoring above cut-off for the SDQ total difficulties score

^4^ Proportions of participants scoring above cut-off for the SDQ lack of prosocial behavior scale

In unadjusted Cox regression models (Table 3), children with identified emotional and behavioural problems were more likely to use habilitation services compared to those with no identified problems. These associations were shown for both mothers’ (HR: 5.02) and fathers’ (HR: 4.25) SDQ ratings. In adjusted cox-regression models, the associations remained significant, although relatively lower HR were obtained for both mothers’ (AHR: 4.24) and fathers’ (AHR: 4.03) ratings. Furthermore, children with identified lack of prosocial behaviour were more likely to use habilitation services than those with no identified problems (AHR: 2.67, AHR: 2.14). Finally, boys were more likely to use habilitation services compared to girls (AHR: 5.34, AHR: 5.08).

**Table 3:** Unadjusted and adjusted cox regression models for exploring the association between emotional and behavioural problems and use of habilitation services. Numbers in bold indicate significant (p < 0.05) associations.

Outcome: Use of habilitation services during follow-up				
	Mothers’ responses (n = 7343)		Fathers’ responses (n = 6322)	
**Independent variables Block I**	**HR (95% Cl)**	**p**	**HR (95% Cl)**	**p**
**Emotional and behavioural problems**				
No identified problems (ref)				
Identified problems^1^	**5.02 (3.31–7.63)**	**< 0.001**	**4.25 (2.81–6.43)**	**< 0.001**
**Independent variables Block II**	**AHR (95% Cl)**	**p**	**AHR (95% Cl)**	**p**
**Emotional and behavioural problems**				
No identified problems (ref)				
Identified problems^1^	**4.24 (2.72–6.61)**	**< 0 .001**	**4.03 (2.64–6.17)**	**< 0.001**
**Lack of prosocial behaviour**				
No identified problems (ref)				
Identified problems^2^	**2.67 (1.52–4.72)**	**< 0.001**	**2.14 (1.26–3.60)**	**0.004**
**Child gender**				
Girl(ref)				
Boy	**5.34 (3.08–9.28)**	**< 0.001**	**5.08 (2.99–8.63)**	**< 0.001**
**Child age**				
5 years (ref)				
4 years	0.86 (0.49–1.51)	0.600	0.73 (0.41–1.29)	0.280
3 years	1.13 (0.69–1.84)	0.630	1.02 (0.63–1.63)	0.940
**Parental country of birth**				
Born in Sweden (ref)				
Born outside Sweden	0.75 (0.41–1.38)	0.360	1.07 (0.61–1.88)	0.820
**Parental education**				
Higher educational level (ref)^3^				
Lower educational level^4^	1.21 (0.79–1.86)	0.380	0.80 (0.52–1.23)	0.310

^1^ Proportions of participants scoring above cut-off for the SDQ total difficulties score

^2^ Proportions of participants scoring above cut-off for the SDQ lack of prosocial behavior scale

^3^College/university degree

^4^ Not completed primary school/completed primary school/completed secondary school

HR = Hazards Ratio, AHR = Adjusted Hazards Ratio, CI = Confidence Interval, Ref = Reference group

## Discussion

This study aimed to investigate the association between early emotional and behavioural problems and use of habilitation services among children in Sweden. The key findings demonstrated that over 30 percent of children using habilitation services had identified emotional and behavioural problems at three to five years of age. In addition, children with these problems, according to both mothers’ and fathers’ SDQ ratings, were more likely to use these services compared to children without such problems. Overall, our results suggest that early emotional and behavioural problems are predictive of use of habilitation services among children in Sweden. This is somewhat expected as older children in the target group of habilitation services, i.e. children with developmental disabilities, display more behavioural problems compared to their typically developing peers [9]. Nonetheless, these problems significantly affect the children’s’ daily lives and development in addition to their developmental disabilities per se [6]. Previous research has proposed that approximately 30–50% of children using similar services as habilitation services also suffer from emotional and behavioural problems, and are thus likely to have mental health treatment needs [27]. Nevertheless, our results show that emotional and behavioural problems were present already at 3–5 years of age and that these early problems predict later use of habilitation services. Further, the SDQ has been shown to predict developmental disabilities such as autism [44, 45] even though this is not the purpose of the instrument. Although some of the SDQ items measure common problems among children with autism, they are not homologous to autism symptoms in general. Thus, using the SDQ as a screening tool specifically for autism could lead to an oversimplification of autism symptomatology, which should be avoided. Rather, the SDQ should be used to measure emotional and behavioral problems, regardless of underlying conditions.

In line with the current findings, a longitudinal study from the Netherlands revealed that parents sought a broader range of support, including child mental health treatment, parental counselling, and specific information for their child’s issues, when their child had both emotional and behavioural problems alongside intellectual disabilities, rather than merely seeking support for intellectual disabilities [46]. As further demonstrated by the results of this study, children with identified lack of prosocial behaviour were more likely to use habilitation services compared to children with no identified problems. In line with our findings, past research has highlighted that parents are inclined to identify early signs of abnormal behaviours in their children when seeking various forms of child mental health interventions, thus, driving their motivation to seek help [47]. Our results, combined with previous research, indicate that families who use habilitation interventions also need support for their child’s emotional and behavioural problems.

Furthermore, in the current study, boys were more likely to use habilitation services compared to girls. This finding is in line with prior research [19]. As demonstrated previously, children who display externalising problems are also more likely to utilise habilitation services, than those without such problems [18]. Given that boys are more likely to exhibit externalising problems compared to girls, boys might be more inclined to consume these services [25, 48]. Our findings, however, revealed that parental socio-demographic factors did not influence use of habilitation services. A plausible rationale supporting this outcome may be that services provided by the CHS in Sweden render information and support to parents regardless of parents’ socio-demographic factors. Nonetheless, the proportions of parents with a high educational level and with Swedish origin were higher in this sample than in the Swedish general population [42]. More parents with lower educational levels and with other origin might have given different results. Additionally, the child’s age was not associated with use of habilitation services. Thus, although the child’s age has been related to habilitation service use in previous research, with younger children more likely to use these services compared to older children [18, 20], the results from this study contradict these findings. Previous studies, however, have included children of a wider age range, i.e., 2–17 years and 6–16 years, than children included in the current study, i.e. 3–5 years. One plausible explanation for our contradictory findings might thus be differences in study populations between the current and previous research.

Overall, our results suggest that early emotional and behavioural problems are important predictors for later use of habilitation services among young children in Sweden. These problems could be identified by using the SDQ within the CHS at the national level. Consequently, this procedure could facilitate delivery of timely interventions to prevent the course of these problems as well as additional negative long-term outcomes. Given the large proportion of children identified with early emotional and behavioural problems among those using habilitation services, habilitation centres could integrate mental health treatment into the standard treatment for children using these services.

### Strengths and limitations

One limitation of this study is the lack of data on specific diagnoses, such as neurodevelopmental disorders, related to the habilitation visits, thus making it impossible to draw a parallel between the disorders and the visits. The parental SDQ ratings can also be considered a limitation due to the risk of over- or under-reporting of the children’s’ emotional and behavioural problems [49]. The analysis relied on cut-off scores for SDQ total difficulties and prosocial behaviour scales, hence overlooking the impact of the remaining SDQ subscales, i.e. emotional symptoms, conduct problems, hyperactivity and peer problems, on habilitation use. Given that the proportional hazards assumption was violated for these scales, they could not be included in the cox regression models. Consequently, further research on the relation between specific emotional and behavioural problems and use of habilitation services is warranted. Furthermore, potential selection bias may exist due to the largely highly educated and Swedish-born parent sample. Thus, generalisations of the findings should be made with caution.

Strengths include the large sample of children and the longitudinal design that followed the children for approximately 6.5 years. Another strength is the use of the SDQ for assessing emotional and behavioural problems, as the instrument has demonstrated acceptable psychometric properties in previous studies on young children [34, 39]. Obtaining separate SDQ ratings from mothers and fathers is considered a strength given that comprehensive information on emotional and behavioural problems were gathered. Data on habilitation services were collected from electronic patient records, thus reducing self-reported bias. Finally, this study fills a research gap by examining the relation between early emotional and behavioural problems and subsequent use of habilitation services among children in Sweden.

## Conclusions

Early emotional and behavioural problems predict subsequent habilitation service use among children in Sweden. Establishing a standardised process to assess emotional and behavioural problems at CHS for all children up to 5 years of age could facilitate early identification and delivery of timely interventions. Due to the large proportion of children identified with early emotional and behavioural problems among those using habilitation services, habilitation centres could integrate mental health treatment into the standard treatment for children using these services.
